# Challenges and perspectives in xenoliver research: lessons from decedent and primate models

**DOI:** 10.3389/frtra.2026.1783796

**Published:** 2026-03-13

**Authors:** Zoe Hahn, Kasra Shirini, Joseph M. Ladowski, Raphael P.H. Meier

**Affiliations:** 1 Department of Surgery, Johns Hopkins University School of Medicine, Baltimore, MD, United States; 2 Department of Surgery, Duke University School of Medicine, Durham, NC, United States; 3 Department of Surgery, University of Maryland School of Medicine, Baltimore, MD, United States

**Keywords:** decedent recipient, liver, living recipient, nonhuman primate, xenotransplantation

## Abstract

Liver xenotransplantation is a potential strategy to address the shortage of donor livers, either as a temporary bridge to allotransplantation, bridge to native liver recovery, or destination therapy. Advances in genetic engineering and immunosuppression have enabled liver xenografts to evade hyperacute rejection, yet durable graft survival remains elusive. Profound thrombocytopenia, consumptive coagulopathy, and xenotransplantation-associated thrombotic microangiopathy limit long-term graft survival. Evidence from nonhuman primates, decedents, and recent clinical models suggests that hematologic and physiologic species incompatibilities (in addition to classical immune-mediated rejection) constitute additional barriers to successful liver xenotransplantation. These problems are driven by species-specific platelet–endothelial interactions, dysregulated coagulation factor synthesis, and platelet sequestration within the xenograft. This review synthesizes lessons from decedent and primate xenoliver models, highlighting progress in this area to date, barriers to prolonged survival after liver xenotransplant, and directions for further research.

## Introduction

Liver transplantation is the only meaningful therapy for end-stage liver disease (ESLD) and faces a growing demand for the limited supply of organs. In 2024, there were 15,025 people added to the liver transplant waitlist, representing a 20% increase from 2020 (1). The increase in demand is likely driven by increases in alcohol- and metabolic-associated liver disease, which has outpaced gains made in the reduction of chronic viral hepatitis-associated liver disease (2). As a result, liver transplant is limited by both a supply of organs which cannot meet demand and the urgency associated with finding and transplanting a matching liver before acute liver failure (ALF) and/or ESLD progresses into multi-organ failure.

Xenotransplantation, specifically the use of a genetically modified pig as a liver donor (i.e., a xenoliver), has long been considered an option to address both issues. For decades, studies in xenolivers were limited by rapid organ failure and rejection but significant advances in genetic engineering have progressed the field to the point of clinical studies in human recipients (3–6). Although the possibility of permanent replacement with a xenoliver is currently out of reach, there are several problems that a xenoliver could address: (1) temporary organ support for ESLD (7) (2) a bridge to allotransplant for those in ALF and (3) a temporizing therapy until organ recovery in acute liver injury (8).

Liver xenotransplantation was first attempted in the 60s and 70s and largely relied on organs from nonhuman primates (NHP). Although NHP xenotransplants benefit from the immunologic, anatomic, and physiologic similarities between NHP and humans, NHPs are unacceptable sources of xeno-organs: they cannot be bred to meet organ demand, are the subject of much ethical debate, and transplanting from NHPs to humans confers considerable infectious disease risk (9). Pigs arose as an alternative organ donor and eventually became the preferred xeno-organ source. Although pigs are immunologically more disparate to humans, they retain anatomic and physiologic similarities, are ethically more acceptable given they are regularly bred and harvested as food, are cheaper to maintain, and provide larger litter sizes after breeding (10, 11).

However, early xenotransplantation experiments with wild-type pig organs resulted in rapid hyperacute rejection due to the immunologic discordance between humans and pigs, leading to graft necrosis and recipient demise within minutes to hours of xenotransplant. Advances in genetic engineering and the development of genetically edited pigs alleviated this concern, opening the door to unprecedented potential in xenotransplantation (3, 12). Nevertheless, many more hurdles remain before the widespread application of clinical xenotransplantation, as will be discussed in this review.

## Anatomical and physiological challenges for liver xenotransplantation

### Before transplant

There are anatomic differences between the pig and human liver that must be considered before transplant, although they do not preclude use of the pig liver as a transplant organ source due to vascular similarities. Namely, the human liver is wedge-shaped and contains two lobes, while the pig liver has four lobes structured in a clover-leaf shape (13), and is smaller on average than a human liver. Both contain eight segments. NHP recipients, which are smaller than human recipients, generally require smaller and younger pig livers than human recipients to optimize anatomic fit (6, 14); whether this may impact functionality of the liver is unclear. Arterial supply, venous drainage, and biliary drainage are generally comparable between the human and pig liver, with minor differences; for example, the arterial supply to the gallbladder in the pig comes from the left hepatic artery pool rather than the right (15). Histologically, pig and human vessels are also similar. The porcine hepatic vein at six months is comparable to the human portal vein in terms of diameter, wall thickness, lumen area, and cross-sectional area, but is shorter in length (16). The porcine portal vein also contains less elastin and collagen, but more smooth muscle, than the human portal vein. Nevertheless, they are equivalent in compliance (17), preventing excessive mechanical force at the site of anastomosis.

Research to date suggests that pig grafts would be physiologically functional in a human transplant recipient. Although comparative studies of pig versus human hepatic vessel pressures are limited, current literature suggests that while portal vein pressure in a pig is equivalent to that in a human, porcine hepatic vein pressure is slightly higher; while hepatic artery resistance is similar, hepatic arterial flow is lower in pigs (18, 19). The impact of differing vessel pressures and flow between a human recipient and pig graft has yet to be clearly delineated. Prior xenoliver experiments have suggested that pig livers are successful at producing and excreting bile (20–22), although this is not always seen (23, 24), possibly due to rapid rejection or liver failure (25). Drug metabolism via the cytochrome P450 system appears similar between pigs and humans, since pig livers are used to model drug clearance (26). Lastly, although pig livers have similar metabolic functionality pertaining to gluconeogenesis as humans, whether they will meet the metabolic demands of a human recipient warrants further study.

### At transplant: coagulopathies

The most pressing question in liver xenotransplantation is whether we can resolve the coagulopathy and platelet dysfunction that is consistently seen in xenoliver experiments. Lethal coagulopathy has been demonstrated in all NHP, decedent, and clinical models of xenoliver transplant to date (6, 20–22). Importantly, this phenotype has persisted despite advances in immunosuppression and genetic engineering, indicating that hematologic failure represents a primary, rather than secondary, barrier to xenoliver survival. *In vitro* studies characterizing thrombocytopenia and thrombotic microangiopathy (TMA) in the xenoliver have suggested multiple etiologies for platelet deregulation.

In a normal physiologic state, the liver synthesizes most clotting factors. In the setting of endothelial injury, glycoprotein Ib binds von Willebrand factor in the endothelium to initiate platelet adhesion and release ADP, which encourages platelet aggregation. Simultaneously, release of tissue factor (TF) from the injured endothelium activates factor VII, which in turn activates factors IX and X, initiating the coagulation cascade. Downstream activation of factor V leads to the formation of thrombin, which in turn converts fibrinogen into fibrin necessary for platelet adhesion to the endothelial wall. On a macro scale, the liver, alongside the spleen, is responsible for continuously sequestering damaged platelets (27).

In a liver xenograft, there is firstly the issue of platelet overactivation and aggregation. *In vitro* studies have evidenced that glycoprotein Ib on primate platelets binds tightly to porcine von Willebrand factor protein (vWF) found on the xenograft's vascular endothelium, causing rapid platelet overactivation and downstream aggregation that is unregulated and takes place without preceding injury to the endothelium (28, 29). This interaction occurs under low-shear conditions characteristic of hepatic sinusoids, making the liver uniquely susceptible to this mechanism. Genetically editing donor pigs to express primate vWF may be a tenable solution to this problem but has not yet been reported and likely has limited clinical utility in human applications (30). Moreover, *in vitro* and *in vivo* studies have shown that porcine endothelium upregulates tissue factor (TF) in the presence of human and NHP plasma, encouraging a pro-thrombotic state (31, 32). This endothelial activation further amplifies coagulation cascade initiation within the xenograft microvasculature, but whether this could be due to an inability of porcine tissue factor pathway inhibitor (TFPI) to inhibit TF is controversial (32, 33).

One NHP study found that serum from baboons with xenolivers took on a clotting factor picture that resembled the composition of clotting factors found in pigs: namely, with lower levels of factors II, VII, and X and higher levels of factors V, VIII, IX, and XI (34). This shift suggests functional dominance of porcine coagulation factor synthesis within the xenograft, which may be pro-thrombotic. Consistent with this finding, it appears that supplementation of preclinical xenoliver recipients with human coagulation factors is protective against thrombosis in the graft (23, 35). However, such supplementation does not fully correct thrombocytopenia or prevent progressive microvascular injury (5). It is unclear whether porcine clotting factors demonstrate diminished functionality in a primate recipient.

Furthermore, liver xenografts consistently sequester primate platelets (28, 34, 36–38) a phenomenon observed across NHP, decedent, and *ex vivo* models, likely due to the ability of liver sinusoidal epithelial cells (LSECs) and Kupffer cells to clear the blood (39). An *ex vivo* model of xenoliver sequestration of human platelets, for instance, found rapid clearance (93% of added platelets were cleared within 15 min) by LSECs (36, 37), demonstrating that platelet sequestration can occur independently of platelet aggregation or immune opsonization. To prevent thrombocytopenia associated with xenoliver transplant, splenectomy is regularly performed, but whether it provides a benefit is unclear (40).

### At transplant: immunologic barriers

Xenoliver experiments using genetically modified pigs have successfully evaded hyperacute rejection (23, 24). Accordingly, immediate antibody-mediated graft loss, once considered inevitable in liver xenotransplantation, is no longer the dominant barrier in contemporary models. As a result of the problem of lethal coagulopathy associated with liver xenografts, long-term data characterizing rejection and immune response in these cases is scarce. Thus, the relative contribution of adaptive immune mechanisms to xenoliver failure remains incompletely defined. However, since the liver functions to produce complement proteins, immunologic crosstalk between a porcine liver NHP primate or decedent model should be anticipated. Importantly, complement-mediated rejection (CMR) has shown to be a formidable cause of rejection in xenotransplantation (41). The liver may be advantaged in this regard**,** since a porcine xenograft will produce its own complement proteins, which are less likely to mediate autologous graft injury (42). The interplay between complement produced by the graft and complement produced by the human recipient in the renal endothelium warrants consideration. Moreover, a native liver in ESLD may not produce enough complement to cause CMR at or near the time of transplant, potentially further attenuating early complement-mediated injury in clinical recipients. This is difficult to assess in a NHP and decedent preclinical model, which would not have ESLD at the time of transplant.

The question then becomes whether porcine complement might pose a risk to human cells and tissues via porcine complement-mediated graft-versus-host-disease (GVHD). Evidence to this point thus far is limited, but there is some data that porcine complement is responsible for human erythrocyte lysis (35). Whether this interaction results in clinically meaningful tissue injury beyond erythrocytes, particularly in the context of liver xenotransplantation, remains unknown.

### After transplant

After xenoliver transplant, the organ must be supported for the length of time required to either bridge to transplant (which could take as little as 24–48 h) (43) or allow for native organ recovery, in which a xenoliver would have to last for weeks or months (44). As is the case in other xenografts, liver xenografts require stringent immunosuppression for the life of the graft. However, given the short duration of survival achieved to date, it remains unclear whether chronic rejection, antibody-mediated rejection, or cellular rejection would ultimately emerge as the dominant failure mechanism once hematologic barriers are sufficiently controlled. Immunosuppression strategies in recent NHP and human experiments are described in Table 1.

**Table 1:** Pig-to-NHP and pig-to-human liver xenotransplants studies and immunosuppression regimen.

Author/center (year)	Donor type	Immunosuppression/other therapies (as reported)	Highest survival (days)
Pig-to-NHP			
Calne et al. (49)	WT	None/AZA + Cs/Cs	3.5
Powelson et al. (50)	WT	Gal depletion, WBI, ATG, pig BMCs	3.1
Hayashi et al. (51)	WT	sCR1	0.21
Ramirez et al. (24)	WT; hCD55	CyP, CsA, Cs	8
Ramirez et al. (52)	WT; hCD55.CD59HT	CyP, CsA, Cs, Daclizumab, RTX, MMF	1
Kim et al. (34)	GTKO, MGH MS	Cs, ATG, Tacrolimus, CVF, AZA, anti-CD154, LoCD2b	9
Yeh et al. (53)	GTKO, MGH MS	Cs, ATG, Tacrolimus, CVF	15
Ji et al. (54)	GTKO, WZ MS	Cs, MMF, ATG, Tacrolimus, CVF, anti-CD154, Dashen	13.6
N-Alvarez et al. (55)	GTKO, MGH MS	Cs, ATG, Tacrolimus, CVF, hPCC (± anti-CD154, LoCD2b depending on subgroup)	7
Shah et al. (56)	GTKO, MGH MS	Cs, ATG, Tacrolimus, CVF, hPCC, Belatacept	25
Shah et al. (23)	GTKO, MGH MS	Cs, ATG, Tacrolimus, CVF, hPCC, anti-CD40mAb	29
Zhang et al. (57)	GTKO WZ MS; GTKO (Bama); GTKO-hCD46	ATG, CVF, Tacrolimus, MMF, Cs	14
N-Alvarez et al. (58)	GTKO	ATG, Tacrolimus, CVF, Cs	11
Lee et al. (14)	DKO2; DKO1; TKO; QKO; TKO + DKI	RTX, ATG, CVF, Etanercept, anti-CD154, Cs, Sirolimus	34
Pig–to-Human			
University of Pennsylvania, United States (47, 59)	69-GE	Steroid only	3 days
Kunming Medical University/Yunnan Agricultural University, China (60)	8-GE	Not reported	Not reported
Tao et al. (20)	6-GE	ATG, Eculizumab, Methylprednisolone, Tacrolimus, MMF, Etanercept, Rituximab	10 days
Zhang et al. (6)	10-GE	Induction: Rituximab, Basiliximab, Steroids; Maintenance: Steroid, Tacrolimus, Sirolimus, MMF	37 days
Xijing Hospital/ClonOrgan, China (61)	6-GE	ATG, Eculizumab, Methylprednisolone, Tacrolimus, MMF, Etanercept, Rituximab	11 days

AHLXT, auxiliary heterotopic liver xenotransplantation; ALS, anti-lymphocyte serum; ATG, anti-thymocyte globulin; AZA, azathioprine; BMCs, bone marrow cells; Cs, corticosteroids; CsA, cyclosporine A; CVF, cobra venom factor; CyP, cyclophosphamide; DKI, double knock-in with human CD46 and human thrombomodulin; DKO1, double knock-out with GGTA1 and CMAH; DKO2, double knock-out with GGTA1 and B4GALNT2; GE, genetically edited; MGH MS, Massachusetts general hospital miniature swine; hPCC, human prothrombin complex concentrate; LoCD2b, rat anti-primate CD2 IgG2b; LXT, liver xenotransplantation; MMF, mycophenolate mofetil; QKO, quadruple knock-out with GGTA1, CMAH, B4GALNT2, and iGb3s; RTX, rituximab; sCR1, soluble complement receptor 1; TMA, thrombotic microangiopathy; TKO, triple knock-out with GGTA1, CMAH, and B4GALNT2; WBI, whole-body irradiation; WT, wild type; WZ MS, Wu Zhanshen miniature swine.

### Long-term outcomes

Currently, long-term outcomes after a liver xenograft are limited in preclinical experiments (6, 20–22). To date, survival in NHP, decedent, and clinical xenoliver models has been insufficient to permit systematic evaluation of chronic graft function or late immunologic complications. More research is needed to set the groundwork for clinical liver xenotransplantation, including understanding how pig coagulation factors and complement proteins will function in a primate recipient, defining the mechanisms underlying xenotransplantation-associated thrombotic microangiopathy, prevention of acute and chronic rejection, and genetically modifying pigs to avoid thrombosis. Importantly, recent human xenoliver experiences suggest that addressing hematologic incompatibility will be a prerequisite to evaluating longer-term immunologic outcomes. Long-term complications, such as the ability of the xenoliver to detoxify, the risk of porcine-derived infectious diseases, the possibility of abnormal folding or storage diseases (45) or the potential for xenograft-related GVHD, are remaining areas of research. Whether prolonged exposure to porcine-derived metabolic and synthetic products is physiologically tolerable in human recipients similarly remains unknown and represents a critical area for future investigation.

## Overview of decedent and primate models

Given the complementary roles of preclinical platforms in advancing liver xenotransplantation, Table 2 provides a comparative overview of the key physiological, translational, and experimental considerations of human decedent versus NHP models. These models address distinct but overlapping questions, and understanding their respective strengths and limitations is essential for interpreting existing data and for guiding future study design.

**Table 2:** Comparison of key physiological study aspects between human decedent versus nonhuman primate (NHP) xenoliver models.

Key physiological/study aspect	NHP model	Decedent human model
Primary purpose and translational role	Mechanistic and interventional platform to test genetics, immunosuppression, and hematologic rescue strategies over longer durations	Near-clinical anatomic scaling and human blood, supports early feasibility, perfusion, short bridging concepts, and human-specific incompatibilities
Baseline recipient physiology (most important limitation)	Also does not reproduce human ESLD and includes species-specific pig-to-NHP incompatibilities that may not mirror pig-to-human	Typically, no sustained ESLD at time of transplant, so does not reproduce the metabolic, immunologic, hematologic milieu of advanced liver failure
Hemodynamics and flow competition	Distinct primate hemodynamics, splenic and platelet biology differences, and potential model-specific flow patterns	Human vascular physiology, but interpretation limited by absence of chronic portal hypertension and ESLD physiology
Early graft function readouts	Similar readouts plus ability to track dynamic physiologic trajectories over time, but in primate rather than human systemic context	Direct demonstration of immediate graft viability: bile output, early synthetic signals (porcine albumin, clotting factors) and short-term perfusion adequacy
Detoxification and metabolic adequacy	Allows longer physiologic observation (relative to decedent) but still may not represent human metabolic demand or ESLD-associated toxin burden	Limited ability to interpret true detoxification requirements and systemic metabolic support needs; short survival windows constrain conclusions
Platelet consumption and sequestration	Robust platform to dissect platelet overactivation, sequestration, and to test interventions (factor replacement, blockade strategies) across time	Human platelets allow direct assessment of human platelet interactions and sequestration tendencies; still limited by short duration
Coagulation factor biology	Historically required complex support (exogenous factors) to extend survival; enables iterative testing of coagulation modification strategies	Human recipient milieu with porcine factor output can be directly assessed, but clinical relevance depends on longer survival and integrated endpoints
xTMA and microvascular injury	Better suited to map onset, progression, and response to targeted interventions, though with primate-specific biases	Can identify emergence signals but typically constrained by short follow-up; difficult to establish full temporal evolution
Complement biology and immune effector balance	Complement pathways are active but pig-to-NHP interactions may differ from pig-to-human; still useful to test complement blockade strategies	Human complement and human antibodies provide high translational relevance; however decedent models lack ESLD-related complement alterations and may not model recipient immunologic fragility
Immune rejection phenotyping over time	Better for sequential histology and immune monitoring, and for defining when adaptive immunity becomes dominant once hematology is controlled	Limited by survival duration; may capture early absence of hyperacute rejection but not chronic phenotypes
Infection and biosafety questions	Enables longitudinal surveillance and immunosuppression-associated infection risk modeling, though not perfectly predictive for humans	Useful for protocolized biosafety workflows and pathogen surveillance signals, but limited for long-term zoonosis assessment
Ability to evaluate long-term complications (GVHD-like phenomena, storage diseases, chronic rejection)	More feasible than decedent models, but still limited by current xenoliver survival ceilings and model-specific incompatibilities	Generally, not feasible because survival is short
Practicality, repeatability, and experimental control	More scalable for repeated mechanistic studies and controlled intervention trials, with the tradeoff of nonhuman physiology	Limited availability, fixed physiology, and fewer intervention cycles; strong human translational relevance for early function metrics
Best-fit questions for the model	Mechanism discovery and optimization: hematologic incompatibility, intervention testing, sequential immune phenotyping, longer-duration support strategies	Human-specific compatibility, early synthetic function and bile production, early complement and coagulation signatures, surgical feasibility

ESLD, end-stage liver disease; GVHD, graft-versus-host disease–like phenomena; NHP, non-human primate; xTMA, xenograft-associated thrombotic microangiopathy.

Recent pig-to-human liver xenograft studies underscore both the excitement and feasibility of xenolivers while providing further information surrounding the remaining barriers. In a 10-day heterotopic auxiliary pig-to-human liver xenotransplantation using a gene-modified donor, the xenograft produced bile early after reperfusion (20). It demonstrated biochemical evidence of hepatic function (including porcine liver–derived albumin), with acceptable vascular flows during the study period. Although thrombocytopenia occurred early, platelet counts ultimately recovered, and histology at study completion showed no clear evidence of rejection, supporting the plausibility of short-term liver xenograft “bridge” support while highlighting hematologic vulnerability as a dominant, unresolved challenge.

Unlike kidney or heart xenotransplantation, liver xenotransplantation is currently envisioned primarily as a temporary, function-supporting therapy, rather than a destination graft. Although modern immunosuppression and genetically edited pigs have addressed the issue of hyperacute and acute rejection, the considerable difficulty in xenoliver survival lies in hematologic dysfunction, as the liver is responsible both for detoxification and the production of coagulation factors (21). Additionally, species-specific incompatibilities in platelet–endothelial interactions and coagulation regulation further exacerbate hematologic instability following xenoliver transplantation. All these factors contribute to poor long-term outcomes. Contrary to the pig-to-NHP xenokidney or xenoheart studies with consistent multi-year survivals, the longest survival in a xenoliver NHP model to date is 34 days as reported by Lee et al. in 2023 (3, 14). This xenoliver experiment was logistically complex as it relied upon not only immunologic blockade, but also the continuous addition of exogenous coagulation factors (14).

An additional barrier made apparent by xenokidney studies was the species incompatibilities unique to pig-to-NHP experiments that may not be seen in pig-to-human studies (46). For this reason, a growing chorus called for human decedent studies to begin. In January 2024, an *ex vivo* pig liver xenograft in a human decedent model was performed, demonstrating early metabolic activity and graft perfusion and approximately 72-hour survival (47). This was followed by a reported 10-day heterotopic auxiliary xenoliver survival in a decedent recipient (20).

Of note, it is important to consider one significant limitation that NHP and decedent models likely do not have long-lasting end-stage liver disease at the time of transplant, as would be the case for a clinical recipient. As a result, these models may incompletely recapitulate the metabolic, immunologic, and hematologic milieu of advanced liver failure. This may make NHP and decedent models non-ideal for the purposes of immunologic (particularly complement-mediated rejection) and hematologic (particularly coagulation factor dysregulation and platelet consumption) study of the xenoliver environment. As a result, further refinement of experimental models will be necessary to generate additional data and support the full transition of xenotransplantation into the clinical realm.

Most recently, Zhang et al. reported a 37-day graft survival following clinical heterotopic xenoliver transplantation into a living patient using a 10-gene–edited pig. This graft was ultimately explanted due to xenotransplantation-associated thrombotic microangiopathy (TMA), rather than classical immune-mediated rejection, consistent with prior findings demonstrating hematologic challenges as the dominant barrier to liver xenotransplantation (6). Importantly, the graft was metabolically active and functioned to secrete bile and clotting factors, including very high levels of factors VIII and IX, with the addition of basiliximab treatment as needed. Platelet counts dropped and fibrinolytic markers stayed elevated after transplant. There was no evidence of hyperacute or acute rejection in this case. The improvement in graft survival in this case relative to that seen in decedent models warrants consideration. Additionally, the immunosuppression protocol used in this case is interesting in that it introduced Basilixumab and did not use complement inhibition within its immunosuppression protocol. It is possible that the elevated complement levels seen throughout the post-op course may have been partially responsible for the development of thrombotic microangiopathy; therefore, more intensive complement modulation might be useful in decedent and NHP translational models.

## Future directions

Hyperacute rejection and rapid graft failure is largely avoided with the use of 10GE pigs and represents a critical prerequisite for clinical translation. However, given the complexity of liver physiology, hematology, and immunology, it is not yet achievable to ensure durable xenoliver function. Unlike renal or heart xenotransplantation, in which rejection is the primary barrier, liver xenotransplantation must first address species-specific incompatibilities. We anticipate that liver xenotransplantation will require further genetic modification of porcine liver grafts to address the hematologic dysfunction that is currently the major limitation to xenoliver experiments. For example, genetically modifying pigs to produce primate clotting factors and primate vWF may be necessary to alleviate platelet activation and aggregation in the xenograft, particularly considering the demonstrated vWF–GPIb mismatch driving platelet overactivation, although the impact on pig survival and development is unclear (48). Similarly, the precise role of porcine LESCs in sequestering primate erythrocytes and platelets, and whether this could be alleviated via genetic modification, has yet to be elucidated (28, 36). Targeted engineering of endothelial regulatory pathways within LSECs may therefore represent a complementary strategy to coagulation factor replacement, though the feasibility and safety of such approaches remain to be determined.

As survival extends beyond the early post-transplant window, the research agenda will broaden from feasibility and acute hematologic stabilization to questions that only emerge with prolonged xenograft function. Longer follow-up will permit systematic assessment of durable synthetic and detoxification capacity, late adaptive immune injury and chronic rejection phenotypes, and the physiologic tolerance of sustained exposure to porcine-derived proteins and metabolites. These improved outcomes will also enable rigorous study of longer-term safety domains highlighted above, including infectious risk, potential xenograft-related GVHD, and unexpected metabolic or storage phenotypes, which remain difficult to evaluate in current short-survival models.

## Conclusions

Xenotransplantation represents a new frontier of transplant medicine facilitated by scientific advancements in genetic editing, immunomodulation, and anatomical considerations. Recent progress in liver xenotransplantation demonstrated that hyperacute rejection can be effectively overcome and that meaningful short-term xenoliver function is achievable. Prior research on liver xenotransplantation using preclinical models provided improved graft survival but was hindered by the considerable platelet and coagulatory dysfunction intrinsic to the liver xenograft at this time. Collectively, available data indicates that hematologic incompatibility, in addition to immune-mediated rejection, currently represents a major barrier to durable xenoliver survival. Although preclinical NHP and decedent models are the best way to study xenotransplant feasibility, safety, and outcomes without risk to a living patient, these models have inherent limitations: the absence of end-stage liver disease, limited survival duration, and species-specific incompatibilities. Future advances in liver xenotransplantation will likely depend on integrated strategies combining further genetic engineering, targeted modulation of coagulation and endothelial pathways, and careful physiologic modeling to enable safe and sustained clinical application.

### Gene-editing definitions

WT (wild type) pigs have no genetic modifications and express all native porcine carbohydrate antigens and regulatory pathways. GTKO pigs have targeted the knockout of GGTA1, thereby eliminating the expression of the *α*-Gal antigen and reducing hyperacute rejection. DKO pigs carry a knockout of GGTA1 and CMAH, removing both α-Gal and Neu5Gc antigens, while TKO pigs additionally lack B4GALNT2, eliminating the SDa antigen; QKO pigs refer to further antigen knockouts built upon the TKO background.

6-GE pigs combine triple knockout of GGTA1, CMAH, and B4GALNT2 with knock-in of selected human complement- and coagulation-regulatory genes, most commonly hCD46, hCD55, and hTBM, to reduce complement activation and early coagulation dysregulation. 8-GE pigs expand on this platform with additional human transgenes targeting innate immune and complement pathways, as reported in early dual-organ xenotransplant feasibility studies.

10-GE pigs are generated on a triple-knockout background (GGTA1, CMAH, B4GALNT2) with knock-in of multiple human complement- and coagulation-regulatory genes, typically including hCD46, hCD55, hCD59, hTBM, hEPCR, and hCD39, to enhance anticoagulant signaling and mitigate xenograft-associated thrombotic microangiopathy.

69-GE pigs represent extensively engineered donors with knockout of GGTA1, CMAH, B4GALNT2, and porcine endogenous retrovirus (PERV) sequences, together with knock-in of multiple human cytoprotective, complement-regulatory, and coagulation-regulatory genes, including hA20, hHO-1, hCD46, hCD55, hCD47, hTBM, and hEPCR, designed to minimize immune injury, inflammation, coagulation dysregulation, and zoonotic risk during xenoliver support.
